# Study on the Mechanism of Flavonoid Enrichment in Black Soybean Sprouts by Abscisic Acid/Melatonin Under Slight Acid Treatment

**DOI:** 10.3390/foods13223567

**Published:** 2024-11-07

**Authors:** Jiyuan Xue, Xiaolan Quan, Jia Yang, Weiming Fang, Yongqi Yin

**Affiliations:** 1 College of Food Science and Engineering, Yangzhou University, Yangzhou 210095, China; mz120232144@stu.yzu.edu.cn (J.X.); mz120211954@stu.yzu.edu.cn (X.Q.); wmfang@yzu.edu.cn (W.F.); 2 Yangzhou Center for Food and Drug Control, Yangzhou 225000, China; jiajia82112001@163.com

**Keywords:** black soybeans, flavonoids, abscisic acid, melatonin, gene

## Abstract

Plant hormones play a critical role in the physiological and biochemical mechanisms of plants, with functions such as regulating the metabolic pathways of secondary metabolite production and alleviating external stresses. In this study, the synthesis of flavonoids in black soybean sprouts was induced by slight acid combined with the plant hormones abscisic acid (ABA) and melatonin (MT). The results indicated that the contents of daidzin, genistin, daidzein, and genistein in black soybean sprouts treated with slight acid were increased by 10 μM ABA and 75 μM MT, and the total flavonoid content was significantly enhanced. Compared with the slight acid treatment, the H_2_O_2_ and malondialdehyde (MDA) contents in black soybean sprouts were increased after ABA treatment, and the black soybean sprouts were further stressed. However, the H_2_O_2_ and MDA contents in black soybean sprouts were significantly decreased after MT treatment, indicating that the stress of black soybean sprouts can be alleviated by MT. Under slight acid stress, the genes related to flavonoid synthesis in black soybean sprouts were induced by exogenous ABA, promoting the accumulation of flavonoids; under exogenous MT treatment, the activity of phenylpropanoid metabolism enzymes was significantly increased, the genes related to flavonoid synthesis were upregulated, and flavonoid synthesis was induced. These results suggest that the combination of slight acid and plant hormone treatments promotes the accumulation of flavonoid substances during the germination of black soybeans. This research lays the foundation for improving the growth conditions of black soybeans and promoting the enrichment of flavonoid substances in black soybeans.

## 1. Introduction

Flavonoids are particularly abundant in leguminous plants. These phenolic secondary metabolites are produced by plants through the phenylpropanoid and flavonoid pathways. Due to their structural similarity to estrogen in mammals, moderate intake of flavonoids can confer multiple health benefits to the human body [1]. Flavonoids play an indispensable role in the prevention and treatment of osteoporosis [2]. For women, postmenopausal discomforts are alleviated by flavonoids [3]. In addition, flavonoids can inhibit the growth of cancer cells [4]. Enhancing the flavonoid content in soybeans and other leguminous crops has, thus, become a focal point of current research.

The quality of black soybeans’ protein (*Glycine max* L.) is excellent, and the content is higher than in regular soybeans [5]. In addition to protein, black soybeans contain a variety of nutrients beneficial to human health, including polyphenols, flavonoids, and other bioactive compounds [6,7,8,9], which have nutritional and health benefits. Bioactive compounds, such as flavonoids, have antioxidant, anticancer, and anti-obesity effects, providing a variety of health benefits to the human body [10]. Compared to other colored soybeans, black soybeans contain more flavonoids and total polyphenols [11], so the antioxidant bioactivity of black soybeans is more prominent.

Our previous research showed that applying slight acid during the germination of seeds can effectively increase the content of flavonoids, but the growth of sprouts will be inhibited [12]. Therefore, we tried to find a method that can both increase the content of flavonoids and alleviate stress. Plant hormones play an important role in plant growth, alleviating stress and promoting plant growth. So, we tried to apply plant hormone treatment on the basis of slight acid treatment to alleviate the stress. In addition, plant hormones play a vital role in the physiological and biochemical mechanisms of plants, and the metabolic pathways responsible for the production of secondary metabolites are regulated by them [13,14]. Abscisic acid (ABA) is a type of sesquiterpene hormone that plays a role in various aspects of plant life. The relative expression levels of *chalcone synthase* (*CHS*) and *Isoflavone synthase* (*IFS*) can be significantly increased by ABA, thereby promoting the increased content of flavonoids in soybeans, according to Jiao et al. [15]. Additionally, melatonin (MT) is a multifunctional biostimulant that plays a crucial role during abiotic stress, mediating reactive oxygen species scavenging and defense systems to alleviate abiotic stress [16], and it also positively influences the biosynthesis of secondary metabolites in plants [17]. Studies have shown [18] that exogenous application of MT effectively mitigates the damage to barley seedlings under NaCl stress by increasing antioxidant enzyme activity and reducing reactive oxygen species. Additionally, the accumulation of phenolic acids in barley seedlings has been induced by enhancing the activity levels of phenylalanine ammonia-lyase (PAL) and cinnamate-4-hydroxylase (C4H). In view of the functions of ABA and MT in promoting the accumulation of plant nutrients and alleviating stress, we attempted to explore whether they are effective in enriching flavonoids in black soybeans and alleviating stress.

Black soybean seeds were used as experimental materials to investigate the effects of ABA and MT on various physiological and biochemical aspects. Furthermore, the impact of ABA/MT on flavonoid metabolism in black soybean sprouts exposed to slight acid treatment has been investigated at the genetic level. New insights into the mechanism of flavonoid enrichment in black soybean sprouts through ABA/MT and slight acid treatment are provided in this study.

## 2. Materials and Methods

### 2.1. Experimental Design

First, 30 g of black soybean seeds was selected, disinfected in a 1% sodium hypochlorite solution for 15 m, soaked in distilled water at 30 °C for 6 h, and then placed in a germinator to sprout in darkness. The temperature of the germinator was controlled at 30 °C. The treated group was sprayed with 30 mL of the treatment solution every 12 h. According to the preliminary experimental results (Figures S1 and S2), we treated the sprouts with 10 μM ABA and 75 μM MT, and took samples for measurement at 12 h and 24 h, respectively.

### 2.2. Extraction, Identification, and Quantification of Isoflavones by HPLC

Daidzein, daidzin, genistein, genistin, and glycitin contents were determined using the method by Tian et al. [19]. In detail, fresh sprouts were added to 80% methanol for grinding, the homogenate was centrifuged at 12,000× *g* for 10 min, and the supernatant was collected. Then, 20 μL of the extraction was analyzed using an Agilent 1200 HPLC system (Agilent Technologies Co., Ltd., Santa Clara, CA, USA). Six isoflavone standards were used for quantifying the isoflavone monomer of black soybean sprouts in HPLC analysis. Identification of the isoflavone was based on comparisons with the retention times of genuine standards. The sample was separated using a ZORBAX SB-C18 column (5 μm particle size, 4.6 × 250 mm; Agilent Technology Co., Ltd., Santa Clara, CA, USA). HPLC parameters: Solvent A, water (0.1% acetic acid); Solvent B, acetonitrile (0.1% acetic acid); elution gradient, the ratio of Solvent B was increased (0–3 min ~ 5–7 min ~ 9–20 min ~ 22–25 min ~ 30 min, 10–15% ~ 30–38% ~ 43–75% ~ 80–75% ~ 10%); detection wavelength, 260 nm; flow rate, 0.8 mL/min; column temperature, 35 °C.

### 2.3. Determination of the Contents of H_2_O_2_, MDA, and $O_{2}^{-.}$

Malondialdehyde (MDA) concentration was determined using the same methodology as that described by Zhuang et al. [20]. A total of 0.5 g black soybean sprouts was ground with 5.0 mL 5% trichloroacetic acid, then centrifuged at 8000× *g* for 10 min. A total of 2.0 mL 0.76% thibabituric acid was added to the supernatant, mixed, and bathed for 30 min. The absorbance of the supernatant was measured at 450 nm, 532 nm, and 600 nm.

The description provided by Zhao et al. [21] was used to calculate the concentration of H_2_O_2_ and superoxide anio ($O_{2}^{-.}$). Take 0.5 g of black soybean sprouts, grind and add to 65 mmol/L phosphate buffer, centrifuge at 8000× *g* for 10min, take 1mL of supernatant, 0.9 mL of 65 mmol/L phosphate buffer (pH7.8), and 0.1 mL of 10 mmol/L hydroxylamine hydrochloride, react at 25 °C for 20 min, add 17 mmol/L p-aminobenzenesulfonic acid and 7 mmol/L α-naphthylamine, react at 25 °C for 20 min, measure the absorbance at 530 nm, and calculate the content in the sample according to the NaNO_2_ standard curve. Take about 0.5 g of black bean sprouts, grind and add 5 mL of 0.1% trichloroacetic acid, centrifuge at 8000× *g* for 10 min. Add 0.5 mL of 0.1% trichloroacetic acid, 10 mM phosphate buffer (pH 7.0), and 1 mL of potassium iodide to 0.5 mL of supernatant, incubate at 28 °C for 1h, and then measure the absorbance at 390 nm.

### 2.4. Total Phenolic Compound Assay

The total phenolic compound was determined according to Limmongkon et al. [22]. Briefly, the reaction was performed by mixing 2 μL of crude extract (10 mg/mL) with 50 μL of Folin reagent, followed by the addition of 50 μL of sodium carbonate (20% *w*/*v*) solution. The reaction was allowed to proceed at room temperature in the dark for 30 min, and then the absorbance was measured at 765 nm. Gallic acid was used as the standard and the result was expressed as μg gallic acid equivalent (GAE)/g dry weight.

### 2.5. Determination of Antioxidant Enzyme Activity

Catalase (CAT) and peroxidase (POD) activity levels were measured as described by Wang et al. [23]. Ascorbate peroxidase (APX) and superoxide dismutase (SOD) activity levels were determined according to Bin et al. [24].

### 2.6. Determination of Antioxidant Capacity

Ferric ion reducing antioxidant potential (FRAP) and 2,2′-azino-bis(3-ethylbenzothiazoline-6-sulfonic acid) (ABTS) were measured with the method used by Rumpf et al. [25].

### 2.7. Determination of the Activity of Metabolic Enzymes

The Wang et al. [26] technique was used to calculate the activity levels of PAL, C4H, and 4CL in cotyledon and non-cotyledon samples. PAL activity was determined directly by spectrophotometric measurement of the conversion of L-phenylalanine to trans-cinnamic acid at 290 nm and was expressed on the basis of fresh weight (U/g FW). The activity levels of C4H and 4CL were measured as the increases in absorbance at 340 nm and 333 nm, respectively, and defined as U/g FW.

### 2.8. RNA Extraction and Quantitative Real-Time PCR Analysis

According to the manufacturer’s recommendations, total RNA was extracted from black soybean sprouts using an E.A.N.A.^TM^ Plant RNA kit (R6827-01, OMEGA, Norcross, GA, USA). Reverse transcription of RNA into cDNA was carried out using the PrimeScript^TM^ RT Master Mix Kit (RR036A, Takara, Japan). Quantitative real-time PCR was performed on the cDNA samples using SYBRR premix EX-Taq™ (RR420A, Takara, Japan). In Supplementary Table S1, the sequence-specific primers designed in the current research are listed.

### 2.9. Statistical Analysis

All results are provided as mean ± standard deviation and each experiment was run in triplicate. Tukey’s multiple range test was used to compare each variable, and a *p*-value of 0.05 was considered significant.

## 3. Results

### 3.1. Effects of Exogenous ABA/MT on the Contents of Flavonoid Monomers in Black Soybean Sprouts Under Slight Acid Treatment

**Figure 1:**
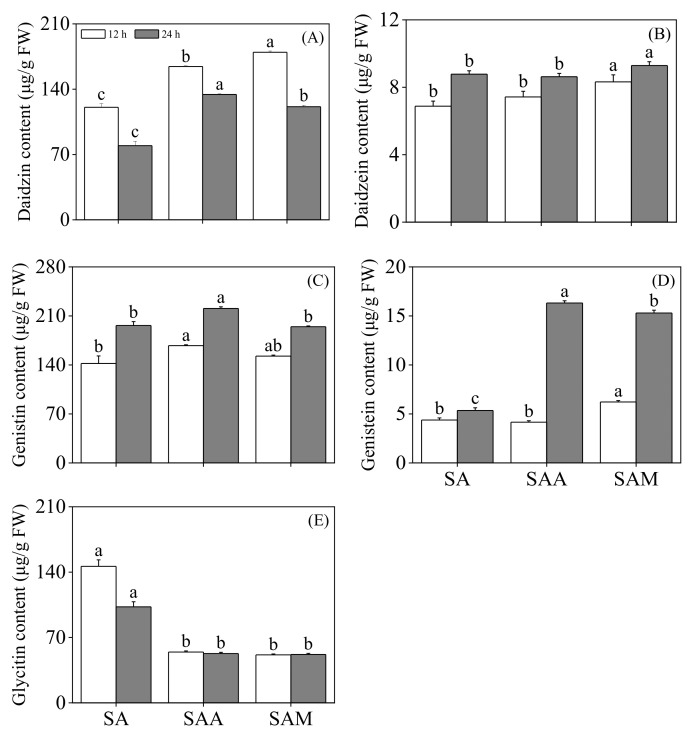
The effect of slight acid treatment combined with ABA/MT on daidzin (**A**), daidzein (**B**), genistin (**C**), genistein (**D**), and glycitin (**E**) in black soybean sprouts. Different lowercase letters indicate significant differences in indicators among treatments at *p* < 0.05 for the same germination time. SA: slight acid; SAA: slight acid + 10 μM ABA; SAM: slight acid + 75 μM MT.

As shown in Figure 1, compared to slight acid treatment, the contents of daidzin and genistin in black soybean sprouts were significantly enhanced by ABA treatment. Specifically, the daidzein content at 12 h and the genistein content at 24 h in black soybean sprouts also increased significantly (*p* < 0.05). However, it is noteworthy that the content of glycitin significantly decreased after ABA treatment (*p* < 0.05). Additionally, the improvement effect of slight acid treatment on the contents of daidzin, daidzein, and genistein in black soybean sprouts was further enhanced with the introduction of MT treatment, while the glycitin content again showed a significant reduction (*p* < 0.05).

### 3.2. Effects of Exogenous ABA/MT on Physiological and Biochemical Parameters of Black Soybean Sprouts Under Slight Acid Treatment

**Figure 2:**
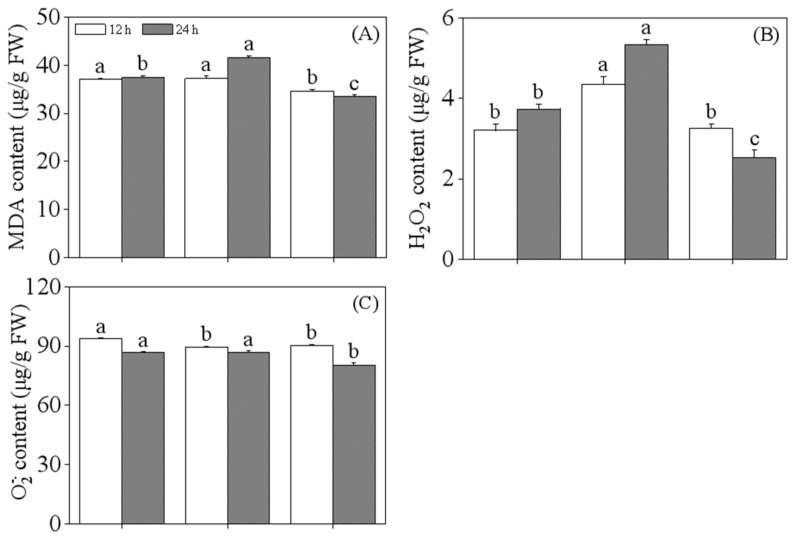
The effect of slight acid treatment combined with ABA/MT on MDA (**A**), H_2_O_2_ (**B**), and $O_{2}^{-.}$ (**C**) in black soybean sprouts. Different lowercase letters indicate significant differences in indicators among treatments at *p* < 0.05 for the same germination time. SA: slight acid; SAA: slight acid + 10 μM ABA; SAM: slight acid + 75 μM MT.

As shown in Figure 2, the H_2_O_2_ content in black soybean sprouts was significantly increased by ABA treatment, and the results after 12 h and 24 h were 1.36 and 1.43 times that of those subjected to single slight acid treatment, respectively. While the MDA content in these black soybean sprouts was significantly elevated (*p* < 0.05) after 24 h, it reached 1.11 times that of the sprouts that underwent single slight acid treatment. This result indicates that ABA treatment induces oxidative stress in black soybean sprouts, leading to the accumulation of H_2_O_2_. Conversely, MDA content was significantly reduced by MT treatment, exhibiting a notable alleviating effect, and H_2_O_2_ content was also significantly reduced in these black soybean sprouts at 24 h (*p* < 0.05).

### 3.3. Effects of Exogenous ABA/MT on Antioxidant Enzyme Activity and Antioxidant Capacity of Black Soybean Sprouts Under Slight Acid Treatment

**Figure 3:**
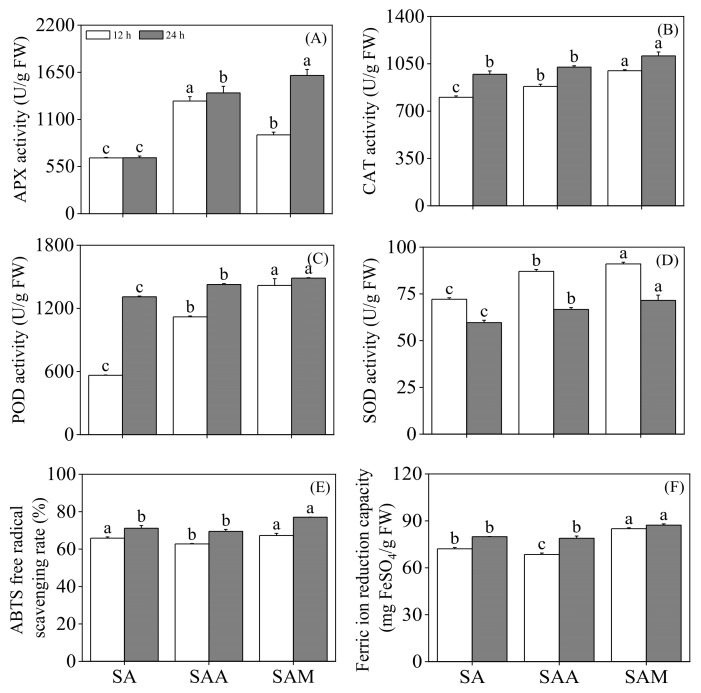
The effect of slight acid treatment combined with ABA/MT on APX activity (**A**), CAT activity (**B**), POD activity (**C**), SOD activity (**D**), ABTS (**E**), and FRAP (**F**) in black soybean sprouts. Different lowercase letters indicate significant differences in indicators among treatments at *p* < 0.05 for the same germination time. SA: slight acid; SAA: slight acid + 10 μM ABA; SAM: slight acid + 75 μM MT.

As shown in Figure 3, the activity levels of APX, CAT, POD, and SOD in the slight acid-treated black soybean sprouts were enhanced by ABA and MT treatments (*p* < 0.05). At 12 h, the APX activity in the ABA-treated black soybean sprouts was the highest, being 2.02 times that of those that underwent slight acid treatment, while at 24 h of germination, the MT-treated black soybean sprouts showed the highest APX activity, which was 2.47 times that of the slight acid-treated sprouts. The CAT activity in the black soybean sprouts at 12 h was significantly increased under ABA treatment. The POD activity in the black soybean sprouts that underwent ABA treatment was continuously enhanced with germination time, while the SOD activity was continuously decreased with germination time in the sprouts that underwent either slight acid treatment or its combination with ABA or MT (*p* < 0.05).

Compared to the slight acid treatment, ABA and MT had different effects on the FRAP capacity and ABTS radical scavenging rate of black soybean sprouts. Under ABA treatment, the FRAP capacity of black soybean sprouts at 12 h was significantly reduced (*p* < 0.05), and the ABTS radical scavenging rate significantly decreased (*p* < 0.05). Under MT treatment, the FRAP capacity of black soybean sprouts was significantly increased (*p* < 0.05), and the ABTS radical scavenging rate of 24-hour germinated black soybean sprouts was significantly elevated (*p* < 0.05) to 1.08 times that of those subjected to slight acid treatment.

### 3.4. Effects of Exogenous ABA/MT on Metabolic Enzyme Activity in Black Soybean Sprouts Under Slight Acid Treatment

**Figure 4:**
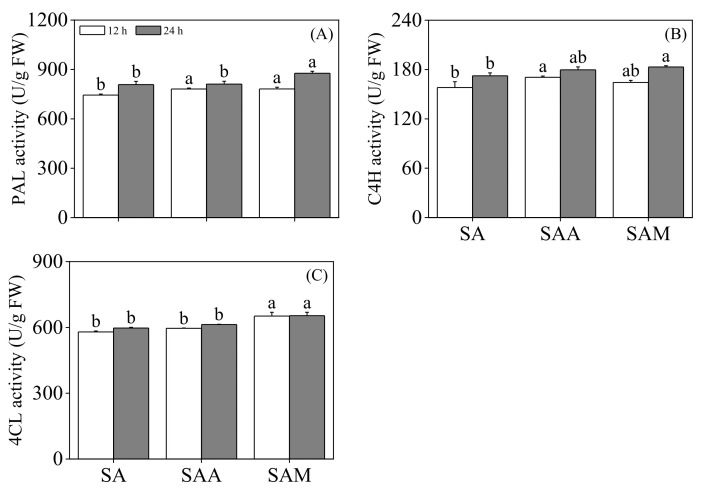
The effect of slight acid treatment combined with ABA/MT on PAL activity (**A**), C4H activity (**B**), and 4CL activity (**C**) in black soybean sprouts. Different lowercase letters indicate significant differences in indicators among treatments at *p* < 0.05 for the same germination time. SA: slight acid; SAA: slight acid + 10 μM ABA; SAM: slight acid + 75 μM MT.

As shown in Figure 4, the activity levels of PAL and C4H in the black soybean sprouts treated with ABA for 12 h were significantly increased (*p* < 0.05) to 1.05 times and 1.08 times that of those that underwent slight acid treatment. Under MT treatment, the PAL activity in the sprouts was notably enhanced (*p* < 0.05) and the C4H activity in the 24-hour sprouts was significantly elevated (*p* < 0.05) to 1.05 times and 1.04 times that of those subjected to slight acid treatment. Additionally, different effects on 4-coumarate: CoA ligase(4CL) activity in sprouts were observed under ABA and MT treatments. Based on slight acid treatment, the activity of 4CL in sprouts was not significantly altered by ABA treatment (*p* < 0.05). However, the 4CL activity in black soybean sprouts was significantly upregulated by MT treatment (*p* < 0.05).

### 3.5. Effects of Exogenous ABA/MT on the Expression Levels of Genes Related to Metabolic Enzymes in Black Soybean Sprouts Under Slight Acid Treatment

**Figure 5:**
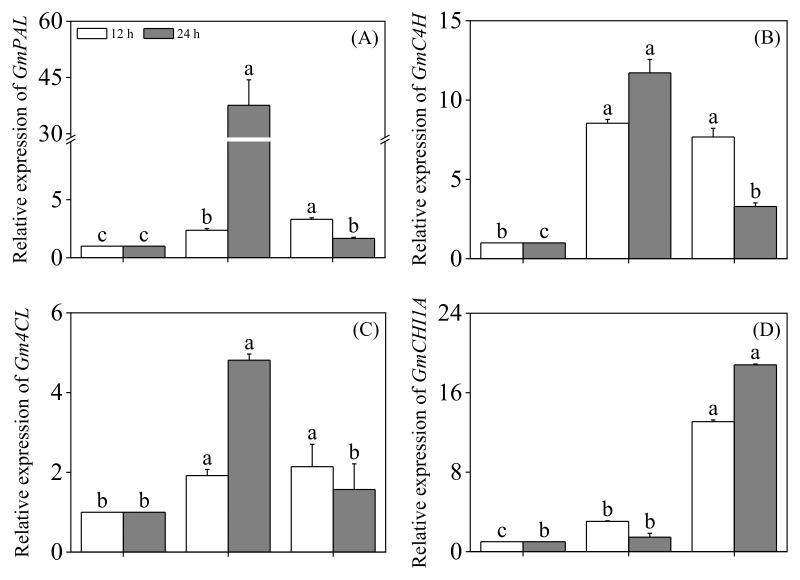

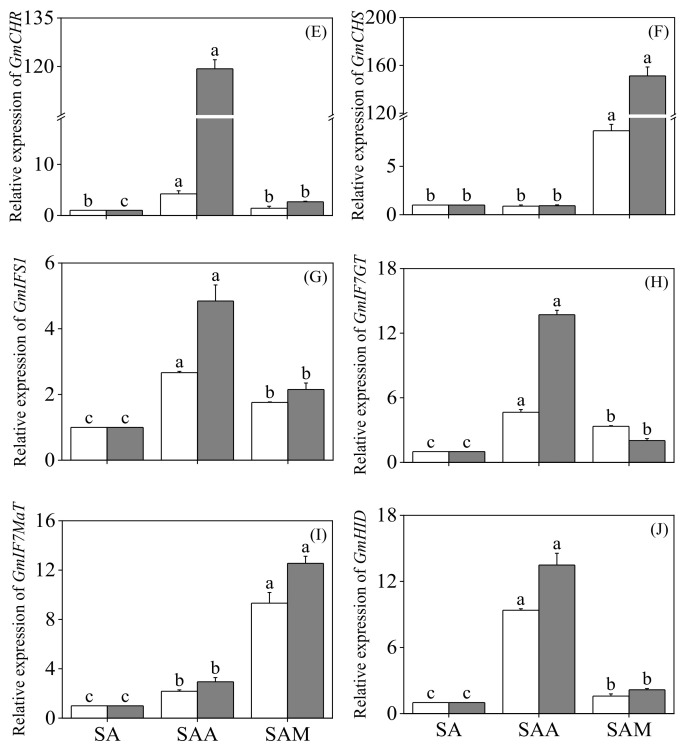
The effect of slight acid treatment combined with ABA/MT on the expression of genes related to metabolic enzymes in black soybean sprouts. Different lowercase letters indicate significant differences in indicators among treatments at *p* < 0.05 for the same germination time. The levels of *GmPAL* (**A**), *GmC4H* (**B**), *Gm4CL* (**C**), *GmCHI1A* (**D**), *GmCHR* (**E**), *GmCHS* (**F**), *GmIFS1* (**G**), *GmIF7GT* (**H**), *GmIF7MaT* (**I**), and *GmHID* (**J**) were measured. SA: slight acid; SAA: slight acid + 10 μM ABA; SAM: slight acid + 75 μM MT.

The changes in the expression levels of genes related to the phenylpropanoid metabolic pathway are shown in Figure 5. Compared to slight acid treatment alone, the expression levels of *GmPAL*, *GmC4H*, *Gm4CL*, *Chalcone reductase (GmCHR)*, *GmIFS1*, Isoflavone 7-O-glucosyltransferase (*GmIF7GT)*, *Isoflavone 7-O-glucoside-6′-O-malonyltransferase* (*GmIF7MaT)*, and *2-hydroxyisoflavanone dehydratase (GmHID)* in 24-hour black soybean sprouts were significantly upregulated by the combined weak acid and ABA treatment (*p* < 0.05). Additionally, the expression levels of *GmPAL*, *GmC4H*, *Chalcone isomerase (GmCHI1A)*, *GmCHS*, *GmIFS1*, *GmIF7GT*, *GmIF7MaT*, and *GmHID* in the 24-hour black soybean sprouts were significantly upregulated by the combined slight acid and MT treatment (*p* < 0.05).

### 3.6. Effects of Exogenous ABA/MT on the Expression Levels of Genes Related to Stress Response and Antioxidant Enzymes in Black Soybean Sprouts Under Slight Acid Treatment

**Figure 6:**
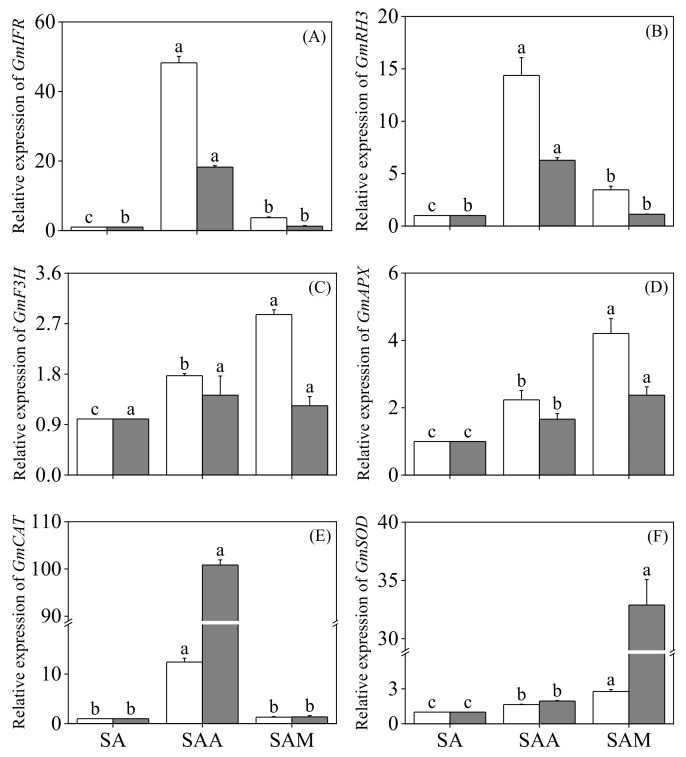
The effect of slight acid treatment combined with ABA/MT on the expression of genes related to stress response and antioxidant enzymes in black soybean sprouts. Different lowercase letters indicate significant differences in indicators among treatments at *p* < 0.05 for the same germination time. The levels of *GmIFR* (**A**), *GmRH3* (**B**), *GmF3H* (**C**), *GmAPX* (**D**), *GmCAT* (**E**), and *GmSOD* (**F**) were measured. SA: slight acid; SAA: slight acid + 10 μM ABA; SAM: slight acid + 75 μM MT.

As shown in Figure 6, compared to slight acid treatment alone, the expression levels of *Isoflavone reductase (GmIFR)*, *RNA helicase gene (GmRH3)*, and *Flavanone 3-hydroxylase (GmF3H)* in 24-hour black soybean sprouts were significantly upregulated by the combined slight acid and ABA treatment (*p* < 0.05). Additionally, the expression levels of *GmIFR*, *GmRH3*, and *GmF3H* in the 12-hour black soybean sprouts were also significantly upregulated (*p* < 0.05).

In the antioxidant system, compared with slight acid treatment alone, the relative expression levels of *GmAPX*, *GmCAT*, and *GmSOD* were significantly upregulated under the combined treatment with slight acid and ABA (*p* < 0.05); the combined treatment using slight acid and MT significantly increased the relative expression of *GmAPX*, and *GmSOD* was upregulated (*p* < 0.05), while the relative expression of *GmCAT* in black bean sprouts under slight acid treatment was not significantly affected by MT.

## 4. Discussion

The polymorphic characteristics and physiological and biochemical indicators of leguminous plants, as well as the contents of the bioactive substances synthesized through their shikimate and phenylpropanoid pathways, can be influenced by germination conditions [27]. When plants are stimulated by external environmental factors, their growth and development are inhibited. To resist and adapt to such adverse conditions, the secondary metabolites in plants undergo significant changes. Abiotic stress is the simplest and most effective method to alter the germination conditions of legumes and promote the production of secondary metabolites [28]. In this study, black soybeans were subjected to slight acid stress during germination, resulting in a noticeable inhibition of sprout elongation and a significant increase in damage-associated indices. Additionally, antioxidant enzyme activity was enhanced, and flavonoid content showed a remarkable increase.

Plant hormones, functioning as signal transducers, can induce the transcription of phenylpropanoid metabolism enzymes, ultimately promoting the accumulation of flavonoid compounds. For instance, the activity levels of enzymes are increased by exogenous ethephon, thus enhancing the accumulation of flavonoid content in soybean sprouts [29]. The flavonoid content in Astragalus has been increased by MeJA treatment [30]. Studies have shown that CHS activity can be reduced by gibberellins, thereby inhibiting flavonoid biosynthesis, while the activity of gibberellin-deficient enzymes is inhibited by abscisic acid, promoting flavonoid biosynthesis. Hence, the addition of higher levels of abscisic acid facilitates the increase of flavonoid compounds. In this study, based on the flavonoid content in black soybean sprouts treated with different concentrations of ABA or MT, it was found that the flavonoid content in black soybean sprouts subjected to slight acid treatment can be further increased by 10 μM ABA or 75 μM MT treatments.

The accumulation of secondary metabolites in most plants is promoted by abiotic stress, during which physiological processes are inhibited, leading to the induction of a significant amount of reactive oxygen species (ROS). ROS are toxic by-products of stress metabolism that cause severe cellular damage through the peroxidation and de-esterification of membrane lipids [31]. The levels of MDA, H_2_O_2_, and $O_{2}^{-.}$ reflect, to some extent, the damage plant cells have incurred. This study found that the levels of MDA and H_2_O_2_ in the germinating 24-hour-old black soybean sprouts increased significantly with the addition of abscisic acid compared to treatment with slight acid alone. However, there was no significant change in $O_{2}^{-.}$ levels. This result indicates that the degree of membrane damage in the black soybean sprouts was exacerbated by ABA treatment. MT is an antioxidant, and one of its most common functions in plants is its role as a protectant, enhancing antioxidants’ capacity to scavenge reactive oxygen species, thereby mitigating various stressors and increasing the plant’s tolerance to external stress [32]. This study found that, compared to slight acid treatment, melatonin treatment alleviated stress in black soybean sprouts, enhancing their tolerance to stress, which was mainly evidenced by significantly reduced levels of MDA, H_2_O_2_, and $O_{2}^{-.}$ during melatonin treatment.

APX, CAT, POD, and SOD are key components of the antioxidant system. Under conditions of oxidative stress, cells activate these components to mitigate the accumulation of reactive oxygen species [33]. Antioxidant enzymes protect plant cells during stress by decomposing H_2_O_2_ into water and oxygen [34]. In this study, compared to those treated with slight acid alone, the activity levels of APX, POD, and SOD in black soybean sprouts significantly increased after the addition of abscisic acid or melatonin, whereas CAT activity showed no significant difference in sprouts treated with slight acid combined with abscisic acid at 24 h when compared to those treated with slight acid alone, but its levels significantly increased under melatonin treatment. Moreover, the relative gene expression levels of APX, CAT, and SOD in black soybean sprouts were significantly upregulated under ABA treatment, whereas APX and SOD were significantly upregulated under MT treatment, with MT having no effect on CAT expression. Despite the increased antioxidant enzyme activity and the significant upregulation of related gene expression under ABA treatment, the H_2_O_2_ and MDA contents in black soybean sprouts remained significantly higher than in those undergoing slight acid treatment. This suggests that the stress damage caused by abscisic acid to black soybean sprouts could not be alleviated by the effects produced by antioxidant enzymes. In this study, the ABTS radical scavenging activity and ferric reducing capability were used to evaluate the effects of different treatments on the antioxidant capacity of black soybean sprouts. The results indicate that, compared to slight acid treatment, the ABTS radical scavenging activity and iron reducing capability of black soybean sprouts cannot be enhanced by combined treatment with slight acid and ABA. However, the iron reducing capability and the ABTS radical scavenging activity of 12-hour black soybean sprouts are significantly improved by combined treatment with slight acid and MT. This conclusion is consistent with the finding that MT reduces H_2_O_2_, MDA, and $O_{2}^{-.}$ in sprouts under slight acidic conditions.

The phenylpropanoid pathway is a key pathway for flavonoid production, with PAL, C4H, and 4CL being the key enzymes in this pathway. To investigate the reasons for flavonoid accumulation, we measured the activity of these key enzymes. Our research indicates that after slight acid treatment for 12 h, the activity of PAL and C4H in sprouts is significantly enhanced by ABA, while the activity of 4CL shows no obvious change. It is possible that abscisic acid promotes the enrichment of flavonoid content in black soybean sprouts through other pathways or enzymes. For example, the research of Ampofo and Ngadi [35] showed that tyrosine ammonia-lyase is also a key enzyme stimulating the production of phenolic substances in the phenylpropanoid pathway. Compared to a control, the flavonoid content in soybean sprouts was increased by enhancing PAL and TAL activity levels through glutamate treatment. Upon ABA treatment, the relative expression levels of *PAL*, *C4H*, and *4CL* were significantly increased, which showed a certain discrepancy with their enzyme activity levels. This may be due to the spatiotemporal asynchrony between gene expression and enzyme activity. This study found that the application of MT after slight acid treatment enhanced the activity of PAL, C4H, and 4CL in black soybean sprouts, and the relative expression levels of these genes were also significantly upregulated compared to the slight acid treatment alone. Vafadar et al. [36] have shown that melatonin can significantly enhance the contents of phenolic compounds in *Dracocephalum moldavica* by markedly increasing PAL activity. Exogenous melatonin promotes the biosynthesis of soybean seedling flavonoids under NaCl treatment by enhancing PAL and 4CL activity [37]. These results indicate that exogenous melatonin can increase flavonoid content by enhancing the activity of key enzymes in the phenylpropanoid pathway.

We further investigated the metabolic pathways of flavonoids and the relative expression levels of relevant genes in downstream metabolic pathways. *CHS*, *CHR*, *CHI1A*, and *IFS1* are closely related to the flavonoid metabolic stage. Devi et al. [38] demonstrated that the upregulation of the relative expression levels of *CHS7*, *CHS8*, *CHR*, and *IFS2* can promote an increase in flavonoid content. Wang et al. [39] showed that the differences in the *CHS1*, *CHS2*, and *CHS3* gene alleles are the principal factors contributing to the variability in flavonoid levels. Hence, the overexpression of these genes facilitates the accumulation of flavonoid content. Jiao et al. [15] showed that abscisic acid treatment increases the relative expression of *CHS* and *IFS*, thereby promoting the soybean flavonoid content. In this study, abscisic acid upregulated the relative expression levels of *CHI1A*, *CHR*, and *IFS1* in slight acid-treated black soybean sprouts, which might be due to the effects of acid treatment. Meanwhile, compared with the slight acid treatment, melatonin increased the relative expression levels of *CHI1A*, *CHS*, and *IFS1* in black soybean sprouts. This indicates differences in the expression of genes involved in the flavonoid biosynthesis pathway during different treatments. The gene *HID* catalyzes the conversion of dihydroflavonoids and flavanol to daidzein and genistein, while *IF7GT* and *IF7MaT* catalyze the glycosylation and malonylation of flavonoid aglycones, respectively, resulting in the production of flavonoid glycosides (daidzin and genistin) and malonyl forms (malonyl daidzin and malonyl genistin) [40]. These results suggest that under slight acid treatment, abscisic acid and melatonin promote flavonoid biosynthesis by upregulating the relative expression of genes related to the downstream pathways of flavonoids.

The genes *IFR*, *RH3*, and *F3H* play a critical role under stress conditions. For example, Cheng et al. [41] believe that *IFR* is unique to the plant kingdom and plays a crucial role in plants’ responses to various biotic and abiotic environmental stresses. Furthermore, *IFR* expression can be significantly increased by abscisic acid. Additionally, *IFR* is involved in regulating the key enzyme for glycyrrhizin synthesis in soybeans. The overexpression of *RH3* facilitates soybean plants’ adaptation to adverse saline conditions [42] and enhances their overall salt tolerance. In this study, the relative expression of *IFR* and *RH3* in black soybean sprouts was significantly upregulated by abscisic acid treatment, with *F3H*’s relative expression increasing after 12 h. Meanwhile, the relative expression of *IFR*, *RH3*, and *F3H* in black soybean sprouts was significantly increased by melatonin after 12 h. This indicates that there are differences in the regulatory effects of abscisic acid and melatonin on stress-related genes in black soybean sprouts undergoing slight acid treatment, and that the growth and the development of black soybean sprouts depend on the combined action of multiple genes.

## 5. Conclusions

Both ABA and MT treatments enhanced the levels of daidzein, genistin, daidzein, and genistein in black soybean sprouts subjected to slight acid treatment, significantly boosting their overall flavonoid content. The ABA treatment elevated H_2_O_2_ and MDA levels in the black soybean sprouts, thereby exacerbating stress conditions, whereas MT treatment markedly decreased $O_{2}^{-.}$, H_2_O_2_, and MDA concentrations, suggesting its potential to mitigate stress in black soybean sprouts. Exogenous ABA stimulated the expression of flavonoid biosynthesis-related genes in black soybean sprouts under slight acid stress, promoting the accumulation of flavonoids. It also increased the activity of antioxidant system enzymes and improved the antioxidant capacity of black soybean sprouts. Conversely, exogenous MT treatment notably enhanced the activity of enzymes involved in phenylpropanoid metabolism, upregulated flavonoid synthesis-related genes, and promoted flavonoid production. This study provides new methods and ideas for improving the growth conditions of black beans, enhancing the antioxidant properties of black beans, and promoting the enrichment of flavonoids in black beans. Black beans treated with plant hormones can be used as ideal functional ingredients or natural antioxidants in new food formulations.

## Data Availability

The original contributions presented in the study are included in the article/Supplementary Materials, further inquiries can be directed to the corresponding author.

## Supplementary Materials

The following supporting information can be downloaded at: https://www.mdpi.com/article/10.3390/foods13223567/s1, Figure S1. The effect of different treatments on flavonoid content in black soybean sprouts. Figure S2. The effect of ABA concentration (I) and MT concentration (II) on flavonoid content in black soybean sprouts undergoing slight acid treatment. Table S1. The sequence-specific primers used in the present study.
