# Supplementary material for: Study on the Mechanism of Flavonoid Enrichment in Black Soybean Sprouts by Abscisic Acid/Melatonin Under Slight Acid Treatment
Source: Foods. 2024 Nov 7;13(22):3567. doi: 10.3390/foods13223567 (PMC11593214; doi:10.3390/foods13223567)
Supplement: Supplementary file 1 [file foods-13-03567-s001.zip › foods-3293022-supplementary.pdf]

Table S1. Primer sequence used in the study. APX: Ascorbate peroxidase, CAT: Catalase, C4H: cinnamate-4-hydroxylase, CHS: chalcone synthase, CHR: Chalcone reductase, CHI1A: Chalcone isomerase, F3H: Flavanone 3-hydroxylase, HID: 2-hydroxyisoflavanone dehydratase, IF7GT: Isoflavone 7-O-glucosyltransferase, IF7MaT: Isoflavone 7-O-glucoside-6"-O-malonyltransferase, IFS1: Isoflavone synthase, IFR: Isoflavone reductase, PAL: phenylalanine ammonia-lyase, RH3: RNA helicase gene, SOD: superoxide dismutase, 4CL: 4-coumarate: CoA ligase.

Gene Name	Forward Primer (5' - 3')	Reverse Primer (5' - 3')
Actin	CTTCCCTCAGCACCTTCCAA	GGTCCAGCTTTCACACTCCAT
GmAPX	CGTGACGATGATTGGGAAGT	TGATAGTGATCTTTCGGACC
GmCAT	ACTACAAATTCTGGTGCTCCTA	TGCAAGCTTCTCCACAAGA
GmC4H	AGGCGAGATCAACGAAGACAAC	GTTCACAAGCTCAGCAATGCC
GmCHS	GCTTGTTGTCTGTTCTGAG	CACCTTCACTGTCTGGAG
GmCHR	CAAAGCCATTGGAGTCAGCAA	CCATGCAAGGTTCATCTCCACT
GmCHI1A	GGCGCTGAATACTCAAAGAAGG	AGAGGCACCAGGTGCAAAATT
GmF3H	TTACCTGGCCCAGGAGAAAAC	ATTCCGGCAAGAGAAATCACTG
GmHID	CACTCTTGCCTGCTCTAAGTT	CTCAACGGTGTGGTGGTAG
GmIF7GT	CCCCACCATTCACCCAAC	TGGCAAGCGTAACTCAAGG
GmIF7MaT	CCCTCTCTTCAAACCTCTCAG	TGGTGGCTTGTTATTCCTATCG
GmIFS1	AGAATTCCGTCCCGAGAGGTT	TGCCATTCCTGAAGTAGCCAA
GmIFR	AGATGGAAATGTGAAAGGAGCG	TGTGCACGGCTTTGTTCAAG
GmPAL	AGCAACACAACCAGGATGTCAA	CAATTGCTTGGCAAAGTGCA
GmRH3	CCTTGAACGTGATGTAGGCTG	GGGCACTTGTTCCTTGTTCTT
GmSOD	TGGTCTCCATGGCTTCCAT	GCTAACGGTACCATCATCA
Gm4CL	AGGCAATGTACGTGGACAAGCT	TCCGAGAGGACAGAGAAGTGGA

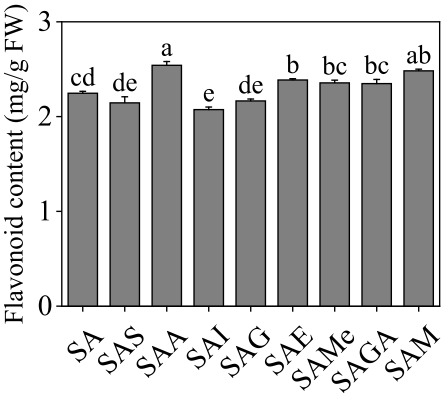

Figure S1. The effect of different treatment flavonoid content in black soybean sprout. SA: slight acid, 126.2 mM pH 5.10 citric acid buffer; SAS: SA + 2 mM salicylic acid; SAA: SA + 15 μM abscisic acid; SAI: SA + 80 μM indoleacetic acid; SAG: SA + 80 μM gibberellin; SAE: SA + 100 μM ethephon; SAMe: SA + 100 μM methyl jasmonate; SAGA: SA + 1 M GABA; SAM: SA + 100 μM melatonin. Lowercase letters reflect the significance of the differences in indexes among treatments at the given germination times using Tukey's test ($p < 0.05$).

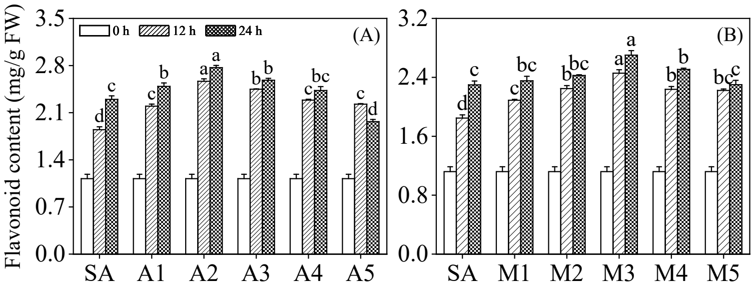

Figure S2. The effect of ABA concentration (I) or MT concentration (II) on flavonoid content in black soybean sprout under slight acid treatment. Lowercase letters reflect the significance of the differences in indexes among treatments at the given germination times using Tukey's test ($p < 0.05$). SA: slight acid, 126.2 mM pH 5.10 citric acid buffer; A1: SA + 5 μM ABA; A2: SA + 10 μM ABA; A3: SA + 15 μM ABA; A4: SA + 20 μM ABA; A5: SA + 25 μM ABA; M1: SA + 25 μM MT; M2: SA + 50 μM MT; M3: SA + 75 μM MT; M4: SA + 100 μM MT; M5: SA + 125 μM MT.
